# Predictors of Successful Weight Restoration in the Treatment of Superior Mesenteric Artery Syndrome: A Systematic Review

**DOI:** 10.3390/nu17182998

**Published:** 2025-09-19

**Authors:** Dennis Gibson, Millie Plotkin, Marina Foster, Philip S. Mehler

**Affiliations:** 1 ACUTE Center for Eating Disorders and Severe Malnutrition at Denver Health, 723 Delaware St., Pav M, Denver, CO 80204, USA; melissamillie.plotkin@dhha.org (M.P.); marina.foster@dhha.org (M.F.); phil.mehler@ercpathlight.com (P.S.M.); 2 Department of Medicine, University of Colorado School of Medicine, Aurora, CO 80045, USA; 3 William B. Glew, MD Health Sciences Library, MedStar Washington Health Center, Washington, DC 20010, USA; 4 Eating Recovery Center, Denver, CO 80230, USA

**Keywords:** SMA syndrome, nutrition, eating disorders, psychological, disorders of gut brain interaction

## Abstract

**Background/Objectives:** Treatment for superior mesenteric artery (SMA) syndrome can include either weight restoration (conservative management) or surgical intervention, with the latter recommended when individuals fail conservative management. However, reasons for failure of conservative management are poorly understood. This systematic review seeks to better understand predictors of treatment outcomes for malnourished individuals with SMA syndrome, specifically regarding weight restoration and behavioral health intervention, and to better understand why individuals fail conservative management. **Methods:** Medline, Embase, and Web of Science were searched for articles that assessed treatment interventions for SMA syndrome in malnourished individuals. **Results:** Seventy-three articles (n = 189 malnourished individuals with SMA syndrome) were included in the final review. Most of the articles (n = 57) had an increased risk of bias as the amount of weight gain with treatment was not explicitly defined and thus the attribution of outcome for “failure” of conservative management could not be ascribed. Modest weight gain (mean 5.64 kg [12.1% body weight increase] or 1.3 kg/m^2^ body mass index increase [9.4% increase in ideal body weight]) was associated with positive outcomes of conservative management. Psychological care also positively impacted treatment outcomes, especially for individuals with comorbid psychiatric conditions. **Conclusions:** Patients who achieve even modest weight gain have resolution of their SMA-related symptoms without a need for surgical intervention. Psychological treatment should be included for any patient struggling to achieve adequate weight restoration, with subsequent improved outcomes, given the high frequency of comorbid mental health illnesses, especially eating disorders.

## 1. Introduction

Superior mesenteric artery (SMA) syndrome is a medical condition associated with abdominal pain and weight loss due to extrinsic compression of the third portion of the duodenum between the SMA and the aorta. Normally, the SMA is positioned such that the duodenum traverses between these vessels unobstructed, with a normal aortomesenteric angle of 38° to 65° and a normal aortomesenteric distance of 10 to 28 mm [1]. However, atrophy of the mesenteric fat pad due to conditions associated with weight loss can potentially result in medial migration of the SMA, with resultant duodenal obstruction. SMA syndrome can rarely also be congenital, can develop in thin adolescents after undergoing surgery for scoliosis, and can develop in adolescents with insufficient weight gain during a growth spurt, amongst other etiologies. This condition is associated with nonspecific gastrointestinal (GI) symptoms that can include postprandial epigastric discomfort/pain, nausea, vomiting, fullness, and heartburn, with subsequent relief of abdominal pain with vomiting. However, radiologic findings do not always correlate with reported symptoms, and differentiation of SMA syndrome from other chronic pain or GI conditions can be difficult [2]. Furthermore, psychological conditions are frequent comorbidities in individuals with SMA syndrome [3], similar to the association between psychological distress and disorders of gut–brain interaction (DGBI) [4].

Diagnosis of SMA syndrome therefore requires a high level of suspicion given the nonspecific symptoms. Computed tomography (CT) scans are considered the gold standard for diagnosis, with an aortomesenteric artery angle ≤25° considered the most sensitive finding, especially with a concomitant aortomesenteric distance ≤8 mm [5]. Other findings can include a dilated proximal duodenum and vertical compression of the third part of the duodenum.

Initial treatment of SMA syndrome should include conservative management with weight restoration, thereby building up the mesenteric fat pad and increasing the angle between the SMA and aorta, which lessens the duodenal obstruction. Furthermore, jejunal feeds, or even consideration of parenteral nutrition (PN) with intolerance of jejunal feeds, should be considered in individuals unable to tolerate oral nutrition [6]. Wan et al. (2020) reported resolution or improvement of GI symptoms in 65% and 15% of individuals with SMA syndrome, respectively, after a mean duration of 24 months of enteral nutrition (EN) [7]. Similarly, Lee et al. (2012) reported successful conservative management in 71.3% of individuals with SMA syndrome [3]. However, no randomized controlled trials or systematic reviews comparing conservative to surgical management have been completed to date. Furthermore, the reasons why patients fail conservative management are poorly understood. The purpose of this study (PICO) is to better understand treatment outcomes in malnourished individuals with SMA syndrome (P), specifically regarding the impact of conservative management and the inclusion of psychological treatment (I) toward resolution of GI distress and/or continued weight restoration (O), in order to better understand why some patients require surgical intervention after failing conservative management (C).

## 2. Materials and Methods

### 2.1. Search Strategy

**Figure 1:**
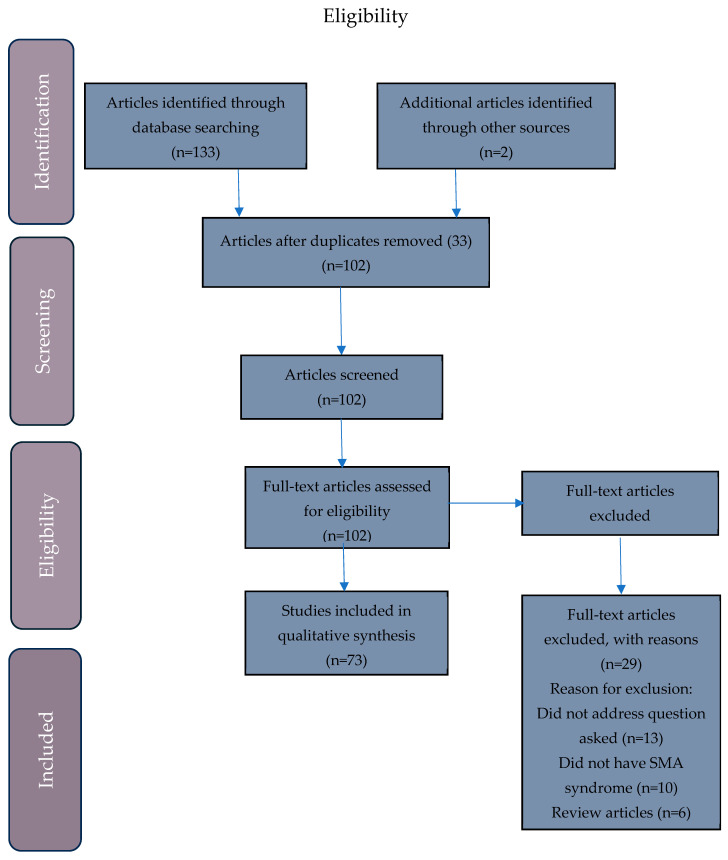
Preferred Reporting Items for Systematic Reviews and Meta-Analysis (PRISMA) flow chart depicting the article selection process.

This study was conducted according to the Preferred Reporting Items for Systematic Review and Meta-Analysis (PRISMA) guidelines [8]. A search of electronic databases (Medline, Embase, and Web of Science) was completed to retrieve all articles published up until 25 August 2025. These databases were searched using the following terms: “superior mesenteric artery syndrome” [All fields] in conjunction with “eating disorders” [All fields] OR “anorexia nervosa” [All fields] OR “bulimia nervosa” [All fields] OR “avoidant restrictive food intake disorder” [All fields] OR “malnutrition” [All fields] OR “severe acute malnutrition” [All fields]. Manual search of articles was also conducted to identify any additional articles that could potentially meet the inclusion criteria. Articles were included if they were published in English and discussed treatment interventions for SMA syndrome in malnourished individuals, defined as a body mass index (BMI) ≤ 18.5 kg/m^2^ or a BMI > 18.5 kg/m^2^ if the study mentioned recent weight loss. Articles were excluded if they were review articles or did not meet the inclusion criteria; there were no exclusions based on the ethnicity, age, or treatment setting of the patient population. The review selection process is summarized in the PRISMA diagram (Figure 1).

### 2.2. Data Extraction and Quality Assessment

The first author extracted relevant data from the included studies using a pre-formulated spreadsheet (see Table S1 for a list of extracted data). If there was any uncertainty about the veracity or relevance of data, the first author consulted with the co-authors. Methodological quality and risk of bias were assessed with the Risk of Bias in Non-randomized Studies—of Interventions, Version 2 (ROBINS-I V2) assessment tool [9]. Important confounding factors to evaluate the risk of bias of the studies for this systematic review included the following questions: Did the patient meet radiologic criteria for SMA syndrome (duodenal obstruction and/or reduced aortomesenteric angle or distance)? Was the amount of weight gained explicitly described?

## 3. Results

### 3.1. Study Selection and Risk of Bias

The literature search resulted in 133 publications, with 2 additional articles identified through additional sources. After removing duplicates and reviewing the titles/abstracts, 102 articles met the inclusion criteria, and the full texts were reviewed. A total of 29 articles were excluded from the systematic review for various reasons, resulting in a total of 73 studies included in the final systematic review (Figure 1). These studies comprised 78 individual case studies, as well as 4 retrospective studies and 1 longitudinal study, for a total of 189 individuals in this entire cohort [10,11,12,13,14,15,16,17,18,19,20,21,22,23,24,25,26,27,28,29,30,31,32,33,34,35,36,37,38,39,40,41,42,43,44,45,46,47,48,49,50,51,52,53,54,55,56,57,58,59,60,61,62,63,64,65,66,67,68,69,70,71,72,73,74,75,76,77,78,79,80,81] (Table S1).

Five studies were considered to have a risk of critical bias due to unclear diagnostic criteria for SMA syndrome (i.e., no mention of either duodenal narrowing/obstruction or the aortomesenteric angle/distance). Fifty-seven of the studies, including the five previously mentioned studies that used unclear diagnostic criteria for SMA syndrome, had an increased risk of bias as the amount of weight gained was not explicitly described. Only 25 case studies and 1 retrospective study were considered to have a low risk of bias.

### 3.2. Demographics and Generalized Outcomes

Table S1 summarizes the findings of the included articles. Patients ranged in age from 5 to 84 years, with a majority of the participants being female (70%). Causes of malnutrition and development of SMA syndrome included eating disorders (n = 37), other medical causes (n = 18), spine surgery (n = 6), religious fasting (n = 2), and unspecified causes (n = 126). Overall, conservative management was associated with a 60% rate of success, and surgical interventions were associated with a 79% rate of success at the follow-up regarding continued tolerance of nutrition and/or resolution of GI symptoms (‘Outcome’ in Table S1). Negative long-term outcomes with regard to nutritional tolerance and/or weight gain were reported in 16 cases (8.5%)—12 individuals did not improve after both conservative and surgical intervention, 1 individual only underwent conservative treatment, and 3 individuals failed surgical intervention without apparent attempts at conservative treatment. Eleven of these cases with negative long-term outcomes were in individuals with an unclear etiology for the development of SMA syndrome, and the other five cases were observed in individuals with eating disorders. Psychological care was mentioned in 22 of the case studies—4 of the 5 individuals who refused psychiatric care as part of the treatment program reported poor outcomes, while all 15 of the individuals who underwent psychiatric care while under the treatment plan reported successful long-term outcomes (two cases did not report on the outcome).

### 3.3. Conservative Management

Initial weight restoration was reportedly successful in 98 (60%) of the case studies, with reported failure in 64 cases (40%) (Table 1). Twenty-one cases were excluded from this analysis due to unclear outcomes. Etiologies of SMA syndrome for the individuals who were successfully treated with conservative management included unspecified (n = 31), eating disorders (n = 21), spinal surgery (n = 5), iatrogenic (n = 2), religious fasting (n = 2), chemotherapy (n = 2), and other causes (n = 5). Forms of nutrition utilized in individuals successfully treated with conservative management included combinations of the following nutritional modalities: oral (n = 29), nasojejunal (NJ) feeds (n = 20), PN (n = 14), unclear (n = 6), nasogastric (NG) feeds (n = 4), and EN without the location specified (n = 1). The total kcal provided was reported for eight cases (range 1500–3000 kcal). Short-term weight gain for individuals who successfully underwent conservative management was reported for 14 individuals, with a mean of 5.64 kg gained (range 1.2–15 kg) over a mean duration of approximately 20 days, resulting in a mean increase in body weight of 12.1% (range 2.8–33%). Watters et al. described a BMI increase of 1.3 kg/m^2^ from baseline (9.4% increase in percentile of ideal body weight) over an average duration of 26.7 days, with resultant resolution or near resolution of associated GI symptoms and tolerance of oral nutrition observed in all patients at discharge [76].

Regarding the 64 individuals who failed conservative management, etiologies for SMA syndrome included unspecified (n = 52), eating disorders (n = 8), and other medical causes (n = 4). Various forms of nutrition utilized in this group included combinations of the following: unclear (n = 32), oral nutrition (n = 15), PN (n = 9), EN without the location specified (n = 5), and NJ feeds (n = 3). Ang et al. utilized a liquid diet in addition to PN or EN for a minimum of six months before considering conservative management a failure [15]. The total kcal utilized was only provided for one individual who failed conservative management (780 kcal) [25]. Chung et al. [21] reported failure of conservative management due to difficulties with the intravenous line utilized for PN, while the other studies alluded to a lack of improvement in clinical symptoms being the reason for failed conservative management. The specific timeline for attempted weight restoration was reported in a limited number of studies as follows: three months of unspecified nutrition [12], six months of a liquid diet in combination with PN or EN [15], four weeks of EN with introduction of orals [24], total PN for nine days [34], unspecified nutrition for two days [73], and a semiliquid diet for four days [73]. Only one study reported no significant change in body weight after attempting an unspecified nutritional intervention for three months [12], while the remainder of the studies failed to explicitly document weight changes prior to a decision to pursue surgical intervention.

### 3.4. Surgical Intervention

Eighty-four individuals underwent surgical intervention, with six case studies excluded from analysis due to unclear outcomes. Surgery was successful in 62 individuals (79%) and unsuccessful regarding continued GI distress and/or an inability to achieve weight restoration in 16 individuals (21%). Sixty-four case studies described patients who underwent duodenojejunostomy, nine individuals underwent gastrojejunostomy, and the remaining studies documented one individual who received mobilization of the ligament of Treitz [48], one individual who underwent infrarenal transposition of the SMA [57], two individuals who underwent Strong’s procedure [61,77], and one individual who underwent an unknown surgical procedure [25]. Negative surgical outcomes, separate from continued GI distress or lack of weight restoration, were reported for seven individuals (8.6%) who underwent surgical intervention, including four deaths [3,31,56,79], two individuals who required lysis of adhesions [15], and abdominal sepsis [23].

### 3.5. Psychological Care

Twenty-two cases described participation (or refusal) of psychological care as part of the treatment plan. Two studies were excluded from the following discussion as clinical outcomes could not be determined. One individual who had intestinal failure and another individual developed SMA syndrome from iatrogenic surgical complications, while the remaining eighteen cases were described in individuals diagnosed with eating disorders. Fourteen individuals who received psychological care achieved successful outcomes--fourteen underwent conservative management and one underwent surgical intervention. The five individuals who refused psychological care comprised one individual with intestinal failure as the cause for SMA syndrome and four individuals with eating disorders. The individual with intestinal failure still achieved successful weight restoration with conservative management, while the other four individuals with eating disorders failed both conservative management and surgical intervention (n = 2), conservative management alone (n = 1), or surgical intervention alone (n = 1).

All 14 individuals with eating disorders who received adequate behavioral health care also reported successful weight restoration. One of these case studies described an individual with GI distress even after duodenojejunostomy, but who experienced significant weight gain with conservative management, upon the inclusion of mental health care into the treatment plan [42]. Similarly, Hundman et al. described a patient who initially struggled with conservative care but experienced resolution of GI distress upon reception of care at a behavioral health facility [33].

### 3.6. Individuals with Eating Disorders

Individuals with eating disorders made up the greatest number of known contributors toward the development of SMA syndrome (n = 37). Diagnoses for these individuals included anorexia nervosa, unspecified subtype (n = 17), the restricting subtype of anorexia nervosa (n = 9), the binge eating/purging subtype of anorexia nervosa (n = 7), bulimia nervosa (n = 1), unspecified feeding and eating disorder (n = 1), and avoidant/restrictive food intake disorder (n = 2). Three cases were ultimately excluded from the following discussion due to unclear outcomes, and a total of 34 individuals were qualitatively analyzed. Successful outcomes were achieved in 70.6% (n = 24) of the cohort regardless of treatment intervention, while 29.4% (n = 10) failed one or both treatment interventions.

Conservative management was successful in 63% (n = 19) of the individuals and unsuccessful in 37% (n = 11). Twelve of these nineteen individuals (63%) who were successfully treated with conservative management received concomitant psychological treatment, while it was unspecified whether the other seven individuals received psychological care. For the individuals who were considered to have failed conservative management, four of these individuals went on to achieve successful symptom control and/or weight restoration with surgical intervention (n = 1, inclusion of psychological care; n = 3, unspecified psychological care), six individuals failed surgical intervention (n = 2 received psychological care; n = 4, unspecified behavioral health intervention), and one individual declined follow-up psychiatric care without surgical intervention.

Four individuals received primary surgical intervention without a clear indication of failed conservative management—all four individuals were unsuccessfully treated with surgery, although one individual thereafter achieved successful conservative intervention with the inclusion of psychological care. Of the other three individuals, psychiatric care was refused by one, and it was unspecified whether the other two individuals received psychological care.

## 4. Discussion

Several findings are suggested from the results of this systematic review. First, the studies which reported “failure” of conservative management alluded to continued GI symptoms as the reason to pursue surgery but without explicitly reporting the actual amount of weight change before surgical intervention. Therefore, the attribution of outcome for “failure” of weight restoration to resolve GI distress could not be ascribed. One can therefore reasonably assume that “failure” in a majority of these cases is more likely due to continued GI distress resulting from a lack of any meaningful weight gain as opposed to continued GI distress in the setting of successful weight gain. Furthermore, the use of NJ feeds or PN, recommended nutritional modalities for individuals with continued GI symptoms presumably due to SMA syndrome, was utilized much less frequently in those who failed conservative management (19%) versus individuals who were successfully treated with conservative management (46%). Similarly, the amount of kcal provided to the individuals who failed conservative treatment was only explicitly reported for one individual (780 kcal) [25], and it was far below the necessary kcal to achieve meaningful weight restoration. On the contrary, the average kcal provided to the cohort of individuals who were successfully treated via conservative management was 2250 kcal (n = 8). Even modest weight restoration—approximately 5.64 kg gained or a 12.1% increase in body weight—was successful at relieving the symptoms of SMA syndrome, presumably via correction of the intestinal obstruction, and with renewed tolerance of oral intake. Thus, the findings of this review suggest that the primary treatment for SMA syndrome should remain nutritional rehabilitation.

It is also suggested that an inability to achieve any meaningful weight gain may be due to psychological comorbidities as a cause for continued GI symptoms. Attention to psychological comorbidities, especially eating disorders, seems to be a critical, yet often overlooked, element in the treatment plan of individuals with SMA syndrome—individuals who received psychological support, regardless of whether surgical or conservative management was pursued, showed improved outcomes compared to those who declined psychological care. Although reverse peristalsis, an infrequently described pathophysiologic finding in SMA syndrome, may contribute to continued GI symptoms, even after surgical intervention, there is a limited understanding of its contribution to GI distress [82,83]. More plausible, Ang et al. judiciously point out that surgical intervention is not guaranteed to cure patients’ symptoms as SMA syndrome mimics many other common GI diseases.

Based on the findings in this systematic review, it would seem that psychological comorbidities and their contribution to GI distress need to be strongly considered before pursuing surgical intervention, as individuals who refused psychological treatment continued to do poorly at the long-term follow up, even after surgical intervention. In the study by Lee et al., the greatest comorbid diagnoses were mental health conditions [3]. Similarly, Sun et al. reported that 50% of the individuals in their cohort who had undergone surgical intervention had comorbid mental health illnesses—symptom improvement was achieved in 11 of these 14 patients, but the remaining 3 patients who experienced continued nausea and vomiting had comorbid psychological conditions of anorexia nervosa, bulimia nervosa, anxiety, and depression [69]. Ang et al. excluded any individuals with eating disorders from their cohort who underwent surgical intervention, achieving 100% treatment success [15]. Indeed, psychiatric diagnoses are highly correlated with DGBIs [84,85]. Curiously, Shen et al. reported significant improvement in obstruction-related symptoms, such as nausea, vomiting, and regurgitation, after surgical intervention for SMA syndrome [86]. However, dyspepsia-related symptoms, such as abdominal pain and bloating, tend to be associated with a functional etiology and were only minimally improved after surgical intervention [87]. This would also support the important distinction between the pathophysiology associated with SMA syndrome and the possible functional symptoms associated with SMA syndrome, similar to the distinction between the pathophysiologic changes in malnutrition and the high prevalence of functional GI symptoms [88]. Treatment for DGBIs, when associated with malnutrition, should include, at a minimum, weight restoration and psychotherapy.

In this review, eating disorders made up the highest number of known direct causes for the development of SMA syndrome. Eating disorders are also commonly associated with many pathophysiologic changes to the GI system along with associated DGBIs [88]. These GI symptoms, regardless of etiology, and other issues specific to eating disorders, make safe weight restoration an arduous albeit critical task in this population. This is due to a number of reasons. First, there is a lack of knowledge for treating individuals with eating disorders due to lack of medical training in this field [89]. Secondly, anorexia nervosa and other eating disorders are egosyntonic illnesses [90], meaning that patients do not desire weight gain or treatment for their malnutrition, and they will often therefore engage in surreptitious behaviors to prevent weight restoration. There also tends to be inherent biases in treating those with eating disorders, which can further impede weight restoration [91,92]. Therefore, achieving successful management of SMA syndrome in those with eating disorders can be problematic, to say the least, if patients are attempting to sabotage any weight restoration.

Limitations of this study include the significant risk of bias in many of the included studies, largely due to weight changes not being explicitly documented. Other limitations include the lack of gray literature in the review, the inclusion of only articles published in English, and not publishing a study protocol before completing the systematic review. Future studies need to explore the amount of weight gain necessary to actually relieve intestinal obstruction, as confirmed by follow-up radiographic imaging, and to better understand whether continued symptoms are related to this obstruction versus a DGBI, although the latter seems more likely based on the findings in this review. Future studies should therefore consider repeat barium studies at incremental weight gains to better understand how quickly duodenal compression resolves.

## 5. Conclusions

Conservative management with weight restoration should always be the primary goal for the treatment of symptomatic SMA syndrome. Patients with significant psychiatric comorbidities, which often contribute to the experienced GI symptoms, seem to benefit from including psychological services, especially with comorbid psychiatric illnesses or the presence of eating disorders. Future studies need to better understand how much actual weight restoration is needed to relieve duodenal obstruction and how much of the symptoms of SMA syndrome are related to this obstruction versus a DGBI.

## Figures and Tables

**Table 1:** Comparison of variables for individuals who achieved successful conservative treatment versus failed conservative management.

Variable	Success	Failure
n (189)	98 (60%)	64 (40%)
Weight gain (mean)	5.64 kg (n = 14)	no significant weight change (n = 1)
	1.3 kg/m^2^ increase in BMI (n = 8)	weight change not specified (n = 63)
Kilocalories prescribed	2250 (n = 8)	780 (n = 1)
Frequency of PO	29 (39%)	15 (23%)
Frequency of PN	14 (19%)	9 (14%)
Frequency of NJ feeds	20 (27%)	3 (5%)
Frequency of EN	1 (1%)	5 (8%)
Frequency of unspecified nutrition	6 (8%)	32 (50%)
Inclusion of behavioral health intervention	14 (100%)	0 (0%)
Individuals diagnosed with eating disorders	19 (63%)	11 (37%)
Received psychological care	12 (63%)	1 (9%)
Declined psychological care	0 (0%)	3 (27%)
Unspecified psychological care	7 (37%)	7 (64%)

Abbreviations: BMI, body mass index; EN, enteral nutrition; NJ, nasojejunal; PN, parenteral; PO, oral.

## Data Availability

Data sharing is not applicable to this article as no new data were created or analyzed in this study.

## Supplementary Materials

The following supporting information can be downloaded at: https://www.mdpi.com/article/10.3390/nu17182998/s1, Table S1: Patient characteristics and outcomes of included studies.
