# Supplementary material for: Predictors of Successful Weight Restoration in the Treatment of Superior Mesenteric Artery Syndrome: A Systematic Review
Source: Nutrients. 2025 Sep 19;17(18):2998. doi: 10.3390/nu17182998 (PMC12472377; doi:10.3390/nu17182998)
Supplement: Supplementary file 1 [file nutrients-17-02998-s001.zip › nutrients-3821200-supplementary.pdf]

**Reference (first author)**	**Study type**	**Age (years)**	**Gender**	**Acute weight loss before diagnosis**	**BMI (kg/m²)**	**Etiology of weight loss**	**Evidence of duodenal obstruction**	**AM angle**	**AM distance**	**Nutritional type (PO, NG, NJ, TPN) and length of time**	**Amount of acute weight gain**	**Reported conservative failure?**	**Surgical intervention**	**Participation in behavioral health treatment?**	**Outcome**	**Judgement (ROBINS-I V2)**
Adson[10]	case study	**35**	F	NOS	NOS	NOS	No	NOS	NOS	NOS	NOS	No	No	NOS	Tolerance of full liquids and minimal abdominal discomfort after 3 days	critical
Albano[11]	case study	**19**	F	10 kg/1 month	NOS	AN	Yes	24°	4 mm	NG feeds x3 days; progressive PO intake thereafter	5 kg/1 month	No	No	NOS	Discharged after 10 days in-hospital with no symptoms	low
Alhadi[12]	case study	**35**	F	NOS	10	AN-BP	Yes	NOS	NOS	NOS (3 months)	No significant change	Yes	GJ	No	Continued refusal to eat after surgery	critical
Aljarad[13]	case study	**35**	M	75 kg/9 months	18.94	Famine from war	Yes	14.6°	3.9 mm	High calorie diet through NJ tube x7 days with progressive oral intake thereafter	NOS	No	No	NOS	Gained 20 kg/3 months without complaints	low
Amano[14]	case study	**12**	F	8 kg/20 months	17.7	NOS	Yes	20°	11 mm	NOS	NOS	No	No	NOS	Able to tolerate normal oral intake on 8th day of hospitalization	critical
Ang[15]	Longitudinal case series (n=12)	**31 (mean)**	4 M/8 F	NOS	18.5 (median)	Psychiatric clearance ruled out active eating disorder, otherwise NOS	Yes	19.4° (mean)	6.2 mm (mean)	5 patients dependent on EN or TPN; nutrition trialed at least 6 months	NOS	NOS	DJ	NOS	Median weight gain of 6.86 kg/3 years; all patients were able to maintain weight without EN or TPN	critical
Bloomberg[16]	case study	**18**	F	6.8 kg/3 months	15.5	AN	Yes	NOS	NOS	PPN x8 days, oral intake was introduced on day 6 with slow advancement over 4 days to a regular diet	NOS	No	No	No	Tolerating some oral intake but having difficulty with weight gain	critical

Reference (first author)	Study type	Age (years)	Gender	Acute weight loss before diagnosis	BMI (kg/m²)	Etiology of weight loss	Evidence of duodenal obstruction	AM angle	AM distance	Nutritional type (PO, NG, NJ, TPN) and length of time	Amount of acute weight gain	Reported conservative failure?	Surgical intervention	Participation in behavioral health treatment?	Outcome	Judgement (ROBINS-I V2)
Bozzola[17]	case study	**13**	F	20 kg/5 months	12.2	AN-R	Yes	23°	3 mm	Combination of PPN (x1 month) with progressive oral and NJ feeds. Final kcal 1800	NOS	No	No	NOS	Received full oral nutrition at 3 months and had gained 11.2 kg	low
Cantella[18]	case study	**39**	F	18 kg/3 years	15.8	NOS	Yes	25°	NOS	Parenteral and enteral (NOS time)	NOS	Yes	DJ	NOS	Gained 10 kg/3 months	critical
Catarino Santos[19]	case study	**39**	F	NOS	NOS	NOS	Yes	11°	4-5 mm	NOS	NOS	Yes	DJ	NOS	Delay in diet acceptance after surgery, and with some weight (not specified) gain at 3 months	critical
Chan[20]	case study	**70**	M	13.3 kg/1 week	18.3	Iatrogenic	Yes	10°	6 mm	NJ feeds x2 weeks. Final kcal 1500	6 kg/2 weeks	No	No	NOS	Reported resolution of SMA syndrome at 1 month based on endoscopy; tolerance of normal diet	low
Chung[21]	case study	**78**	F	NOS	19	Parkinson's disease	Yes	NOS	NOS	difficulties with TPN due to intravenous line placement (NOS time)	NOS	Yes	GJ	NOS	BMI increased to 22.5 kg/m² at 3 months	critical
Civan[22]	case study	**16**	F	15 kg over undefined time period	14.7	NOS	Yes	5°	NOS	NJ feeds	NOS	No	No	NOS	Tolerated enteral nutrition with weight gain	moderate
Datey[23]	case study	**60**	M	NOS	NOS	NOS	Yes	23°	12 mm	IV alimentation (NOS time)	NOS	Yes	DJ	NOS	Developed abdominal sepsis after surgery but discharged home without duodenal obstruction at 3 weeks	critical
de Rodriguez[24]	case study	**21**	F	NOS	16.9	NOS	Yes	13°	3.3 mm	NJ and PO feeds. Final kcal ~2900	3 kg/6 weeks	No	No	NOS	Continued to gain weight on follow-up (BMI 18.7 kg/m²) with lack of GI symptoms	low

Reference (first author)	Study type	Age (years)	Gender	Acute weight loss before diagnosis	BMI (kg/m²)	Etiology of weight loss	Evidence of duodenal obstruction	AM angle	AM distance	Nutritional type (PO, NG, NJ, TPN) and length of time	Amount of acute weight gain	Reported conservative failure?	Surgical intervention	Participation in behavioral health treatment?	Outcome	Judgment (ROBINS-I V2)
	case study	**17**	F	NOS	8.3	AN	Yes	23°	7.4 mm	Progressive enteral nutrition (up to 38 kcal/kg [780 kcal] with introduction of orals on day 21 (4 weeks)	NOS	Yes	DJ	NOS	Required two procedures due to PO intolerance after first procedure; NJ tube placed after 2nd procedure due to suboptimal PO intake. Thereafter received oral intake with outpatient team (BMI 13 kg/m^2)	critical
de Silva[25]	case study	**28**	F	NOS	NOS	AN-BP	Yes	NOS	NOS	NOS	NOS	NOS	NOS	NOS	Continued food refusal and emesis after surgery	critical
Diaz-Martinez[26]	case study	**32**	M	42 kg/2 years	NOS	Complications of diabetes	Yes	17°	5.3 mm	Parenteral nutrition without improvement in oral feeding tolerance (NOS time)	NOS	Yes	DJ	NOS	Recovered 50% of the lost body weight at 5 months	critical
Elbadawy[27]	case study	**18**	F	NOS	12.7	AN	Yes	NOS	NOS	NOS	NOS	Yes	GJ	No	Weight gain of 0.9 kg/couple months; Similar abdominal complaints experienced 6 months after surgery	critical
Foster[28]	case study	**18**	M	NOS	NOS	triple A syndrome	Yes	NOS	NOS	Underwent Heller's myotomy for achalasia with improvement in oral feeding tolerance	NOS	NOS	No	NOS	BMI improved to 17.8 kg/m^2 at age 19	low
Fraser[29]	case study	**32**	F	13.6 kg/6 months	NOS	NOS	Yes	NOS	7.2 mm	NOS	NOS	NOS	DJ	NOS	Tolerance of liquids on post-operative day 1 with advancement to pureed foods; denied symptoms and tolerated regular diet at follow-up	critical

Reference (first author)	Study type	Age (years)	Gender	Acute weight loss before diagnosis	BMI (kg/m²)	Etiology of weight loss	Evidence of duodenal obstruction	AM angle	AM distance	Nutritional type (PO, NG, NJ, TPN) and length of time	Amount of acute weight gain	Reported conservative failure?	Surgical intervention	Participation in behavioral health treatment?	Outcome	Judgement (ROBINS-I V2)
Froese[30]	case study	**16**	M	40% weight loss/6 months	NOS	NOS	Yes	NOS	NOS	NOS	NOS	No	No	Yes	"Condition improved" without providing specifics	critical
Genser[31]	case study	**38**	.	NOS	12.3	tuberculosis	Yes	11.5°	4 mm	NOS	NOS	NOS	GJ	NOS	Death	critical
Gwee[32]	case study	**17**	F	10 kg/1 year	16.4	AN-R	Yes	NOS	NOS	NJ feeds with transition to orals	NOS	No	No	Yes	Gained 12.9 kg while on the eating disorder unit	low
Hundman[33]	case study	**15**	F	9 kg/4 months	NOS	ARFID	NOS	NOS	NOS	Started on tube feeds with transition to TPN and transition back to NG feeds	NOS	No	No	Yes	Lost 6.3 kg on home NG feeds; thereafter received follow-up care at behavioral health facility with successful completion of treatment	low
Jacobs[34]	case study	**55**	M	16 kg/2 weeks	19.4	thyrotoxicosis	Yes	21°	5 mm	TPN (x9 days)	NOS	Yes	DJ	NOS	Achieved BMI of 23 kg/m² over next 8 months	critical
Johnson[35]	case study	**19**	F	27.7 kg/1 year	19.1	drugs/AN	Yes	8°	6 mm	NG feeds with progression to PO on hospital day 4	2.3 kg/1 week	No	No	NOS	NOS	low
Jordaan[36]	case study	**13**	F	NOS	11.9	AN	NOS	NOS	NOS	NOS	NOS	Yes	DJ	No	Abdominal symptoms returned after surgery, and weight was unchanged at 2 years	critical
Khanal[37]	case study	**65**	M	NOS	NOS	NOS	Yes	35°	3.4 mm	NOS	NOS	NOS	DJ	NOS	Normal state of health at subsequent follow-ups	critical

Reference (first author)	Study type	Age (years)	Gender	Acute weight loss before diagnosis	BMI (kg/m^2)	Etiology of weight loss	Evidence of duodenal obstruction	AM angle	AM distance	Nutritional type (PO, NG, NJ, TPN) and length of time	Amount of acute weight gain	Reported conservative failure?	Surgical intervention	Participation in behavioral health treatment?	Outcome	Judgement (ROBINS-I V2)
Kim[38]	case study	**31**	M	9 kg/18 days	19.4	Iatrogenic	Yes	NOS	7 mm	TPN x2 weeks with continued weight loss and with subsequent addition of jejunal feeding (final kcal 1900)	6 kg/2 weeks	No	No	No	Relief of symptoms	low
Kirby[39]	case series	**17**	M	NOS	14.5	NOS	Yes	34°	3.5 mm	NOS	NOS	NOS	DJ	NOS	Tolerance of normal diet and BMI increased to 19.1 kg/m^2	critical
		**45**	F	NOS	19	NOS	Yes	18°	7 mm	NOS	NOS	NOS	DJ	NOS	Continued postprandial discomfort at 29 weeks postoperative with unchanged BMI	critical
		**21**	F	NOS	15.7	NOS	Yes	NOS	7 mm	NOS	NOS	NOS	DJ	NOS	Initial resolution of symptoms but vomiting returned at month 7 and BMI was 15.8 kg/m^2 at 62 weeks	critical
		**69**	F	NOS	16.2	NOS	Yes	NOS	8 mm	NOS	NOS	NOS	GJ	NOS	Full recovery noted at 4-week follow up and with resolution of GI symptoms but with a BMI of 15 kg/m^2	critical
Kornmehl[40]	case study	**16**	F	9 kg/few months	11.7	NOS	Yes	NOS	NOS	Oral liquid feeding supplying up to 2500 kcal daily with gradual introduction to a soft diet	10 kg/few weeks	No	No	NOS	Gained an additional 14 kg/2 months without GI distress	low
Kumar[41]	case study	**.**	M	NOS	NOS	Religious fasting	Yes	8-9°	2-3 mm	NG feeding x1 week with tolerance of regular diet thereafter	NOS	No	No	NOS	Remained asymptomatic at 6 months	critical

Reference (first author)	Study type	Age (years)	Gender	Acute weight loss before diagnosis	BMI (kg/m²)	Etiology of weight loss	Evidence of duodenal obstruction	AM angle	AM distance	Nutritional type (PO, NG, NJ, TPN) and length of time	Amount of acute weight gain	Reported conservative failure?	Surgical intervention	Participation in behavioral health treatment?	Outcome	Judgement (ROBINS-I V2)
Kurisu[42]	case study	**18**	F	16.5 kg/2 months	10.9	AN-BP	NOS	NOS	NOS	"dietary nutrition and central venous nutrition"	NOS	No	No	Yes	History of DJ but remained symptomatic; BMI increased to 13.5 kg/m² after 70 days of conservative treatment	critical
Laffont[43]	case study	**54**	M	12 kg/3 months	NOS	Spinal surgery	Yes	NOS	NOS	NJ nutrition and parenteral nutrition with transition to PO feeding at 2 weeks	8 kg/2 weeks	No	No	NOS	Gained 12 kg/1 month; discharged at 5 months without GI distress	low
Lee[3]	Retrospective chart review (n=73)	**28 (median)**	27 M/53 F	NOS	17.4 (median)	Comorbid mental disorders in 21.3% of population	Yes	10.5° (median)	5 mm (median)	TPN indicated in 51 patients; no other information provided	NOS	22% of patients failed	GJ, DJ, and Strong's procedure	NOS	71.3% of patients successfully treated with medical management, 12% of patients managed surgically without pre-trial of medical management, and 22% of patients considered "failure" with medical management	critical
Lippl[44]	case study	**20**	M	10 kg/12 months	19.3	NOS	Yes	NOS	7 mm	parenteral nutrition	NOS	No	No	NOS	Gained 3 kg/3 months and reported relief of symptoms	low
	case study	**31**	M	7 kg/3 years	17.3	Chemotherapy	Yes	NOS	6 mm	parenteral nutrition	NOS	No	No	NOS	Gained 5 kg/2 months	low
Lo[45]	case study	**26**	F	6.4 kg/1 year	16	AN	Yes	NOS	NOS	full liquid diet with tolerance of 1500 kcal	NOS	Yes	No	NOS	Presented to another hospital 2 weeks after discharge with BMI of 15.9 kg/m² and underwent DJ but continued to lose weight (BMI of 12.4 kg/m² at 6 weeks postoperative)	critical

Reference (first author)	Study type	Age (years)	Gender	Acute weight loss before diagnosis	BMI (kg/m²)	Etiology of weight loss	Evidence of duodenal obstruction	AM angle	AM distance	Nutritional type (PO, NG, NJ, TPN) and length of time	Amount of acute weight gain	Reported conservative failure?	Surgical intervention	Participation in behavioral health treatment?	Outcome	Judgement (ROBINS-I V2)
Maraqah[46]	Case study	**16**	F	7 kg/4 years	9.8	NOS	Yes	18.2°	NOS	NOS x6 weeks	NOS	Y	GJ	NOS	Weight increase of 8 kg over next 4 months with resolution of symptoms	critical
Mascolo[47]	case study	**47**	F	NOS	10.6	AN-BP	NOS	22°	4 mm	Tolerated pure liquid diet and remained symptom free	NOS	No	No	NOS	Started gaining weight and was symptom free	moderate
Mearelli[48]	case study	**47**	M	NOS	NOS	NOS	Yes	13.4°	7.7 mm	Parenteral nutrition but with continued symptoms (NOS timeline)	NOS	Yes	Mobilization of ligament of Treitz	NOS	Doing well at 6 months	critical
Moreno[49]	case study	**14**	F	5 kg/4 weeks	(86% IBW)	spinal fusion surgery	Yes	NOS	NOS	Failed to gain weight with 2 weeks of NJ feeds but achieved 2.5 kg weight gain on TPN with progression to tolerance of PO food	2.5 kg/2 weeks	No	No	NOS	Continued to achieve slow weight gain with home PO feeds	low
Neto[50]	case study	**45**	F	7 kg/5 days	18	severe diarrhea	Yes	NOS	NOS	Initiation of TPN (NOS timeline)	NOS	NOS	DJ	NOS	Gained 12 kg/18 months	critical
Ojemolon[51]	case study	**26**	M	5.5 kg/3 weeks	18.2	Religious fasting	Yes	20°	6 mm	NJ feeds with transition or soft diet	2.3 kg/9 days	No	No	NOS	Remained symptom free with continued weight gain	low
Pannu[52]	case study	**69**	F	NOS	17.1	NOS	Yes	21°	6 mm	NOS	NOS	No	No	NOS	NOS	critical

Reference (first author)	Study type	Age (years)	Gender	Acute weight loss before diagnosis	BMI (kg/m^2)	Etiology of weight loss	Evidence of duodenal obstruction	AM angle	AM distance	Nutritional type (PO, NG, NJ, TPN) and length of time	Amount of acute weight gain	Reported conservative failure?	Surgical intervention	Participation in behavioral health treatment?	Outcome	Judgement (ROBINS-I V2)
Park[53]	case study	**29**	F	NOS	17.65	BN	Yes	7°	NOS	Soft diet	NOS	No	No	NOS	Remained well at 1 week	critical
Pathak[54]	case study	**14**	M	NOS	NOS	tuberculosis	NOS	5°	NOS	NOS	NOS	No	No	NOS	Improved symptoms with weight gain	moderate
Pentlow[55]	case study	**21**	F	35 kg/18 months	NOS	AN	Yes	NOS	NOS	NOS	15 kg/1 month	No	No	NOS	Gained an additional 7 kg/6 months	low
Poudel[56]	case study	**64**	M	NOS	NOS	NOS	Yes	20°	NOS	NOS	NOS	NOS	DJ	NOS	Death	critical
Pourhassan[57]	case study	**37**	F	8 kg/unknown time	16	NOS	Yes	14°	NOS	NOS	NOS	Yes	Infrarenal transposition of the SMA	NOS	Remained in good physical condition at 9 months without mention of weight trends	critical
Rao[58]	case study	**13**	F	9.4 kg/4 months	15.8	Chemotherapy	Yes	16°	NOS	NJ feeds and TPN	Gain of 1.2 kg	No	No	NOS	Continued weight gain, reaching the 80th percentile	low
Recio-Barbero[59]	case study	**38**	M	NOS	18.3	USFED	NOS	9.2°	NOS	No weight gain with PO and intolerance of NJ feeds. Patient subsequently reported volitional vomiting and subsequently gained weight as EN was transitioned to PO	8.8 kg/6 weeks	No	No	Yes	Continued eating disorder care with weight stability at BMI ~20 kg/m^2	low
Record[60]	case study	**13**	F	NOS	NOS	NOS	Yes	15°	NOS	Gained weight with TPN with control of symptoms over the next year but required follow up	NOS	Yes	DJ	NOS	Tolerance of regular diet but no mention of weight at 1 year	critical

Reference (first author)	Study type	Age (years)	Gender	Acute weight loss before diagnosis	BMI (kg/m^2)	Etiology of weight loss	Evidence of duodenal obstruction	AM angle	AM distance	Nutritional type (PO, NG, NJ, TPN) and length of time	Amount of acute weight gain	Reported conservative failure?	Surgical intervention	Participation in behavioral health treatment?	Outcome	Judgement (ROBINS-I V2)
	case study	**16**	F	NOS	NOS	AN	Yes	NOS	NOS	hospitalizations for exacerbation of symptoms<br>NJ feeds without resolution of symptoms with weight fluctuations between 33 and 47 kg	NOS	Yes	DJ	NOS	Tolerance of regular diet with weight stability at 2 weeks	critical
Rehman[61]	case study	**15**	F	NOS	NOS	AN	Yes	NOS	NOS	NOS	NOS	Yes	Strong's operation	NOS	Weight gain of 4 kg/couple weeks	critical
Sahni[62]	case study	**54**	F	NOS	15	NOS	Yes	NOS	NOS	Intolerant of NJ feeds x2 weeks with transition to TPN for NOS time	NOS	Yes	DJ	NOS	Tolerance of oral feeds without GI distress	critical
Seo[63]	case study	**14**	F	25 kg/6 months	11.09	NOS	Yes	9.1°	NOS	Soft diet	2.1 kg/16 days	No	No	NOS	Multiple hospital readmissions due to intentional weight loss and binge eating	low
Shintani[64]	case study	**41**	F	NOS	14	AN	Yes	NOS	NOS	TPN	NOS	No	No	NOS	Maintained her weight around BMI 19.3 kg/m^2 at 4 years follow-up	low
Shreyas[65]	case study	**37**	F	4 kg/5 months	18.1	NOS	Yes	12.9°	4.5 mm	NOS	NOS	NOS	DJ	NOS	Discharged postoperative day 10	critical
Singh[66]	case study	**26**	F	NOS	16.2	AN-R	NOS	NOS	NOS	NOS	NOS	No	No	NOS	Weight gain and resolution of symptoms with 3 weeks of nutritional rehabilitation	critical
Sinwar[67]	case study	**37**	M	15 kg/4 months	NOS	NOS	Yes	NOS	NOS	TPN (NOS time)	NOS	NOS	DJ	NOS	Discharged on postoperative day 7 with no complaints of vomiting and tolerance of semi-solids	critical
Sours[68]	case study	**17**	F	35 kg/6 months	NOS	AN	Yes	NOS	NOS	TPN x1 week with eventual tolerance of PO	NOS	No	No	NOS	Tolerance of oral feeds	critical

**Reference (first author)**	**Study type**	**Age (years)**	**Gender**	**Acute weight loss before diagnosis**	**BMI (kg/m²)**	**Etiology of weight loss**	**Evidence of duodenal obstruction**	**AM angle**	**AM distance**	**Nutritional type (PO, NG, NJ, TPN) and length of time**	**Amount of acute weight gain**	**Reported conservative failure?**	**Surgical intervention**	**Participation in behavioral health treatment?**	**Outcome**	**Judgement (ROBINS-I V2)**
Sun[69]	Retrospective chart review (n=14)	**39 (mean)**	3 M/11 F	10.7 kg (mean)/unknown time	19.9 (mean)	50% of patients with comorbid mental health illnesses	Yes	NOS	NOS	Use of EN required in 3 patients	NOS	Yes	DJ	NOS	At mean follow up of 20 months, durable symptom improvement achieved in 79% of patients with mean BMI gain of 1.38 kg/m^2 for all patients	critical
Tharu[70]	case study	**32**	F	9.1 kg/3 months	19.5	NOS	Yes	15.4°	NOS	Clear liquid diet with transition to solids	NOS	No	No	NOS	Clinically improved	critical
Tsirikos[71]	Retrospective chart review (n=4)	**13 (mean)**	F	NOS	17.8 (n=1)	2.4% of adolescents who underwent corrective surgery for scoliosis developed SMA syndrome	Yes	NOS	NOS	NJ feeds	NOS	Yes (n=1)	Derotation of duodenum and jejunum	NOS	All patients doing well at 2-3 years	critical
Van Rensburg[72]	case study	**73**	M	NOS	16.2	post hip replacement	Yes	23°	8.5 mm	NJ feeds with increase in PO intake as tolerated	NOS	No	No	NOS	Gained 5 kg/3 months	low
Vannatta[73]	case series	**17**	F	30.5 kg/6-8 months	NOS	AN	Yes	NOS	NOS	NOS x2 days	NOS	Yes	DJ	NOS	Gained 10 kg/2 weeks and remained symptom free at 1 year follow-up	critical
		**21**	F	NOS	NOS	NOS	Yes	NOS	NOS	Semiliquid diet x4 days	NOS	Yes	DJ	NOS	No recurrence of symptoms in the following 1.5 years	critical
		**5**	F	1.4 kg/2 weeks	NOS	NOS	Yes	NOS	NOS	PO liquids	NOS	No	No	NOS	No further symptoms at 1.5 year	critical
Verhoef[74]	case study	**16**	F	22 kg/16 months	15	AN-R	Yes	NOS	NOS	NJ feeds + PO (total kcal of 2900) with	NOS	No	No	Yes	14 kg gained/1 year	low

Reference (first author)	Study type	Age (years)	Gender	Acute weight loss before diagnosis	BMI (kg/m^2)	Etiology of weight loss	Evidence of duodenal obstruction	AM angle	AM distance	Nutritional type (PO, NG, NJ, TPN) and length of time	Amount of acute weight gain	Reported conservative failure?	Surgical intervention	Participation in behavioral health treatment?	Outcome	Judgment (ROBINS-I V2)
										transition to all PO 3000 kcal diet						
Vethakkan[75]	case study	**19**	M	20 kg/2 years	10.04	Hypothalamic germinoma	Yes	16°	NOS	Parenteral nutrition + NJ feeds (2.5 weeks)	6.7 kg/10 days	No	No	NOS	NOS	low
Watters[76]	Retrospective chart review (n=8)	**33 (mean)**	F	NOS	12.95 (mean)	AN	Yes	NOS	NOS	3 patients treated with jejunal feeds + PO; 5 patients treated with liquid diet (one transitioned to solids)	Mean increase in BMI of 1.3 kg/m^2 over average of 26.7 days	No	No	Yes	6 patients with resolution of symptoms and 2 patients with improvement in symptoms	low
White[77]	case study	**84**	M	13.6 kg/2 years	20.96	NOS	Yes	NOS	NOS	NOS	NOS	NOS	Strong's operation	NOS	Tolerating diet without symptoms and positive weight gain	critical
Yao[78]	case study	**17**	F	10 kg/3 years	14	AN	Yes	10°	5.5 mm	NOS with five hospitalizations over three years	NOS	Yes	DJ	Yes	Gain of 3 kg/2 months	critical
Yap[79]	case series	**58**	F	NOS	NOS	ESRD	Yes	NOS	NOS	Did not tolerate NJ feeds due to repeated malpositioning of the feeding tube; subsequently started TPN (NOS time)	NOS	Yes	GJ	NOS	Remained dependent on TPN due to continued symptoms	critical
		**46**	M	NOS	NOS	ESRD	Yes	NOS	NOS	NOS	NOS	NOS	GJ	NOS	Death	critical

Reference (first author)	Study type	Age (years)	Gender	Acute weight loss before diagnosis	BMI (kg/m²)	Etiology of weight loss	Evidence of duodenal obstruction	AM angle	AM distance	Nutritional type (PO, NG, NJ, TPN) and length of time	Amount of acute weight gain	Reported conservative failure?	Surgical intervention	Participation in behavioral health treatment?	Outcome	Judgement (ROBINS-I V2)
		**68**	F	NOS	NOS	ESRD	Yes	NOS	NOS	Enteral feeding	NOS	No	No	NOS	Death	critical
Yazdani [80]	case study	**18**	F	6.82 kg/unknown time	14.76	ARFID	Yes	NOS	NOS	PO nutrition	NOS	No	No	No	NOS	critical
Yi[81]	case study	**12**	M	8.5 kg/2 years	10.76	Intestinal failure	Yes	NOS	NOS	Combination of TPN and PO with increase in PO intake	NOS	No	No	Yes	Gained 11 kg/10 months	low

Table S1. Patient characteristics and outcomes of included studies

Abbreviations: AM (aortomesenteric), AN (anorexia nervosa), AN-BP (anorexia nervosa, binge eating/purging type), AN-R (anorexia nervosa, restricting type), ARFID (avoidant/restrictive food intake disorder), BMI (body mass index), BN (bulimia nervosa), DJ (duodenojejunostomy), EN (enteral nutrition), ESRD (end stage renal disease), F (female), GJ (Gastrojejunostomy), IV (intravenous), kg (kilogram), M (male), NG (nasogastric), NJ (nasojejunal), NOS (unspecified), PO (per oral), PPN (peripheral parenteral nutrition), SMA (superior mesenteric artery), TPN (total parenteral nutrition),
